# Präoperative Strahlentherapie von retroperitonealen Weichteilsarkomen: Ergebnisse der EORTC-STRASS-Studie

**DOI:** 10.1007/s00066-021-01753-w

**Published:** 2021-03-04

**Authors:** Ann-Kristin Kalisch, Andreas Dunst, Jürgen Dunst

**Affiliations:** Christian-Albrechts-Universität zu Kiel, Kiel, Deutschland

## Hintergrund

Sarkome sind eine überaus heterogene Gruppe mit zahlreichen histologisch und biologisch unterschiedlichen Subtypen. Retroperitoneale Sarkome gehören mit einer Inzidenz von 0,5 bis 1 pro 100.000 zu den seltenen Erkrankungen [8]. Der Stellenwert der Operation ist unstrittig. Die Prognose hat sich in den letzten 15 Jahren verbessert [5]. Als wesentliche Faktoren für diese positive Entwicklung werden vermehrt Therapien an spezialisierten Zentren sowie Fortschritte in der Op.-Technik und im perioperativen Management angenommen [2, 4, 6]. Bezüglich adjuvanter Therapien war die Datenlage spärlich und kontrovers. Im Jahr 2016 fanden Nussbaum und Mitarbeiter in einer Analyse von fast 10.000 Patienten der National Cancer Database (NCDB) einen Überlebensvorteil von 10 %-Punkten für die zusätzliche prä- oder postoperative Radiotherapie im Vergleich zur alleinigen Operation [7]. Diese Daten bedingten bisher die beste verfügbare Evidenz zum Stellenwert der (neo-)adjuvanten Strahlentherapie. In einem Editorial zu der Nussbaum-Arbeit wurde schon 2016 auf die laufende EORTC-62092-Studie hingewiesen [1]. Deren Daten wurden jetzt publiziert, zeigen jedoch nicht den erwarteten Vorteil durch die Strahlentherapie [3]. Die Studie wurde bereits im Märzheft in unserer Zeitschrift vorgestellt und mit einem Literaturkommentar kritisch gewürdigt (G.G. Grabenbauer, Strahlenther Onkol 196, März 2020). Jetzt möchten wir hier einige Details ergänzen, die für interdisziplinäre Diskussionen im Tumorboard wichtig sein könnten.

## Patientenkollektiv und Methodik der EORTC-62092-Studie

Die multizentrische randomisierte Studie schloss Patienten ein mit histologisch gesichertem unifokalem retroperitonealem Sarkom ohne Fernmetastasen. Der Tumor durfte weder über das Zwerchfell noch die Incisura ischiadica major hinausgehen. Die Patienten mussten ≥ 18 Jahre alt sein, einem WHO Performance Score von ≤ 2 haben und sowohl für eine Strahlentherapie als auch für die Operation geeignet sein. Ausgeschlossene histologische Subtypen waren gastrointestinale Stromatumoren, Rhabdomyosarkome, primitive neuroektodermale Tumoren oder andere klein-, rund- und blauzellige Tumoren, Osteosarkome, Chondrosarkome, die aggressive Fibromatose sowie sarkomatoide oder metastatische Karzinome. Die Patienten wurden je nach Therapiearm entweder mit einer neoadjuvanten Strahlentherapie (50,4 Gy in 28 Fraktionen, fünfmal pro Woche) und anschließender Operation oder lediglich mit Operation behandelt. Primärer Endpunkt war das abdominal rezidivfreie Überleben. Sekundär wurden das Tumoransprechen auf die präoperative Strahlentherapie, das metastasenfreie Überleben, das abdominal rezidivfreie Intervall, das Gesamtüberleben und die therapiebedingten Nebenwirkungen analysiert.

## Ergebnisse

Zwischen Januar 2012 und April 2017 wurden 266 Patienten (133 je Arm) an 31 Institutionen in Europa, Kanada und den USA rekrutiert. Die mediane Nachbeobachtungszeit betrug 43,1 Monate. 128 des Op.-Arms und 119 des Radiotherapie-plus-Op.-Arms erhielten die geplante Therapie. Folgende histologische Typen waren häufig: Liposarkome (ca. 75 %), Leiomyosarkome (ca. 15 %) und andere. In keinem der Endpunkte zeigte sich für die Gesamtgruppe ein Vorteil durch die Strahlentherapie. Das 3‑Jahres-Gesamtüberleben betrug 84,6 % in der Op.-Gruppe und 84 % in der RT-plus-Op.-Gruppe. Als häufigste Nebenwirkung vom Grad 3–4 in der RT-plus-Op-Gruppe zeigte sich eine Lymphopenie, welche bei 77 % der behandelten Patienten dieser Gruppe auftrat und nur bei einem Patienten in der Op-Gruppe.

## Schlussfolgerung der Autoren

Eine präoperative Bestrahlung sollte ab sofort nicht mehr generell empfohlen werden. Es ist jedoch nötig, weiterhin mehr Daten zu diesem Thema zu sammeln.

## Kommentar

Mit der ersten randomisierten Phase III-Studie zum Thema Bestrahlung beim retroperitonealen Sarkom, einer seltenen Erkrankung mit einer anspruchsvollen Therapie, hat die Studiengruppe eine beachtliche Leistung erbracht. Ihre Qualität zeichnet sich auch durch eine hohe Rate an protokollgerechten Behandlungen aus (93 %), was für die therapeutische Expertise der teilnehmenden Institutionen spricht. Die Aussage dieser Studie mit der momentan höchsten Evidenz ist somit als richtungsweisend hinsichtlich der Behandlungsempfehlungen für die lokale Therapie von retroperitonealen Sarkomen zu verstehen.

In einer Post-hoc-Analyse, in der der Progress unter neoadjuvanter Bestrahlung in Kombination mit einer makroskopisch vollständigen Resektion nicht als Endpunkt gewertet wurde, lag das abdominal rezidivfreie Überleben nach 3 Jahren bei 58,7 % (95 %-KI 49,5–66,7) in der Op.- und 66 % (57,1–73,5) in der RT-plus-Op.-Gruppe. In der größten Subgruppe, den Liposarkomen, zeigte sich in der Post-hoc-Analyse ein Trend zu einem besseren abdominal rezidivfreien Überleben durch Radiotherapie (65,2 % in der Op.-Gruppe vs. 75,7 % in der RT-plus-Op.-Gruppe, HR 0,62, n. s.).

Zwei wichtige Aussagen der Studie waren für die bislang etablierte Strahlentherapie negativ. Erstens zeigte sich kein Überlebensvorteil durch die Strahlentherapie, nicht einmal im Trend. Zweitens gab es auch im primären Endpunkt der abdominalen Rezidivfreiheit keinen signifikanten „benefit“ durch die zusätzliche Strahlentherapie. Eher zeigte sich ein höheres Nebenwirkungsprofil, insbesondere für Lymphopenien im Strahlentherapiearm. Jedoch zeigen sich nach sorgfältiger Analyse der Details und der im Supplement publizierten zusätzlichen Daten doch einige Hinweise auf einen positiven Effekt der Strahlentherapie versteckt: Erstens, zusammenhängend mit der Definition des primären Endpunkts, ergab sich hier nämlich überhaupt kein Vorteil für die Strahlentherapie, da in mehreren Fällen lokale Progressionen bereits unter der neoadjuvanten Strahlentherapie vor der Op. aufgetreten waren (Größenzunahme nach RECIST). Das betraf 15 Patienten, immerhin also mehr als 10 % der RT-plus-Op-Gruppe. Diese Patienten konnten alle komplett R0 reseziert werden, und 4 von ihnen (27 %) entwickelten später ein Lokalrezidiv; das entspricht etwa der Lokalrezidivrate des Studienarms mit alleiniger Op. Die Strahlentherapie scheint also diesbezüglich nicht nachteilig gewesen zu sein. Wenn man diese Patienten (wie in der Post-hoc-Analyse gemacht) nicht als Progression werten würde (weil sie ja durch die anschließende Op. kontrolliert wurden), ergibt sich dann ein Trend zugunsten einer besseren lokalen Kontrolle durch die Strahlentherapie. Zweitens zeigte sich in der Subgruppe der Liposarkome (welche etwa drei Viertel aller Patienten ausmachte) ein Trend für ein besseres lokalrezidivfreies Überleben. Nach der Analyse des Independent Data Monitoring Committee, das allerdings nur im „supplementary appendix“ enthalten ist, wird unser Befund sogar noch deutlicher. Denn es traten ja bei den Liposarkomen Lokalrezidive bei 32/100 Patienten nach der Op. und bei 14/98 Patienten nach RT-plus-Op. auf. Drittens trennen sich die Kaplan-Meier-Kurven der Lokalrezidivfreiheit mit zunehmender Nachbeobachtungszeit immer deutlicher, vor allem bei den Liposarkomen. Die absolute Differenz – im Text nicht ausdrücklich erwähnt, aber aus den Kurven ablesbar – beträgt in der Lokalrezidivfreiheit etwa 5 %-Punkte nach einem Jahr, etwa 10 %-Punkte nach 3 Jahren und mehr als 20 %-Punkte nach mehr als 5 Jahren. Die Hazard Ratio für die Verhinderung von Lokalrezidiven bei Liposarkomen ist mit 0,62 durchaus respektabel, erreichte aber formal keine Signifikanz.

Die Konsequenz dieser Studie ist somit, dass die (präoperative) Strahlentherapie für dieses Kollektiv keinen Vorteil bringt und unterlassen werden sollte. Das heißt aber nicht, dass man im Einzelfall nicht trotzdem eine Strahlentherapie diskutieren sollte.

## Fazit

Vor allem gibt EOTRC 62092 Hinweise auf einen „benefit“ durch die Strahlentherapie in der Subgruppe der Liposarkome, und hier insbesondere der Low-grade-Liposarkome. Hierzu folgt inzwischen eine weitere Studie der EORTC-Studiengruppe. Für die klinische Praxis bleibt festzuhalten, dass es bei Vorliegen von Weichteilsarkomen mit hohem Lokalrezidivrisiko auch weiterhin gute Argumente für eine prä- bzw. postoperative Strahlentherapie gibt.

*Ann-Kristin Kalisch und Andreas Dunst, Kiel*
